# Markov Switching Model for Quick Detection of Event Related Desynchronization in EEG

**DOI:** 10.3389/fnins.2018.00024

**Published:** 2018-02-01

**Authors:** Giuseppe Lisi, Diletta Rivela, Asuka Takai, Jun Morimoto

**Affiliations:** ATR Computational Neuroscience Laboratories, Department of Brain Robot Interface, Kyoto, Japan

**Keywords:** Markov switching model, Bayesian estimation, quickest detection, event related desynchronization, sensorimotor rhythms, electroencephalogram, neuroprosthesis, brain computer interface

## Abstract

Quick detection of motor intentions is critical in order to minimize the time required to activate a neuroprosthesis. We propose a Markov Switching Model (MSM) to achieve quick detection of an event related desynchronization (ERD) elicited by motor imagery (MI) and recorded by electroencephalography (EEG). Conventional brain computer interfaces (BCI) rely on sliding window classifiers in order to perform online continuous classification of the rest vs. MI classes. Based on this approach, the detection of abrupt changes in the sensorimotor power suffers from an intrinsic delay caused by the necessity of computing an estimate of variance across several tenths of a second. Here we propose to avoid explicitly computing the EEG signal variance, and estimate the ERD state directly from the voltage information, in order to reduce the detection latency. This is achieved by using a model suitable in situations characterized by abrupt changes of state, the MSM. In our implementation, the model takes the form of a Gaussian observation model whose variance is governed by two latent discrete states with Markovian dynamics. Its objective is to estimate the brain state (i.e., rest vs. ERD) given the EEG voltage, spatially filtered by common spatial pattern (CSP), as observation. The two variances associated with the two latent states are calibrated using the variance of the CSP projection during rest and MI, respectively. The transition matrix of the latent states is optimized by the “quickest detection” strategy that minimizes a cost function of detection latency and false positive rate. Data collected by a dry EEG system from 50 healthy subjects, was used to assess performance and compare the MSM with several logistic regression classifiers of different sliding window lengths. As a result, the MSM achieves a significantly better tradeoff between latency, false positive and true positive rates. The proposed model could be used to achieve a more reactive and stable control of a neuroprosthesis. This is a desirable property in BCI-based neurorehabilitation, where proprioceptive feedback is provided based on the patient's brain signal. Indeed, it is hypothesized that simultaneous contingent association between brain signals and proprioceptive feedback induces superior associative learning.

## 1. Introduction

Sensorimotor rhythm-based brain robot interfaces (BRI) have recently gathered attention in the field of neurorehabilitation (Daly and Wolpaw, 2008). In this context, rehabilitation is conducted by activating a device that assists movement using the brain signal. The assisted movement produces sensory input that is hypothesized to induce central nervous system (CNS) plasticity, leading to the restoration of normal motor control. Endogenous brain computer interfaces (BCI), such as the one based on sensorimotor rhythms (SMR), have been used in motor neurorehabilitation as an effective tool for promoting neuroplasticity of neuromuscular pathways (Silvoni et al., 2011).

SMR are modulated by movement or motor imagery, and they are often referred to as event-related (de)synchronization (ERD/ERS). They can be detected by non-invasive methods such as the electroencephalogram (EEG). An ERD is a power decrease of mu (7–13 Hz) and beta (13–30 Hz) rhythms that occur in the sensorimotor areas during a motor-related task, while an ERS is a power increase following the offset (i.e., end) of the task (Pfurtscheller and Lopes da Silva, 1999). The ERD/ERS elicited by motor imagery have been used to control neuroprosthetic devices such as functional electrical stimulation (FES) to achieve the hand motion in spinal chord injury (Müller-Putz et al., 2005) or stroke patients (Daly et al., 2009), and to activate upper (Gomez-Rodriguez et al., 2011; Ramos-Murguialday et al., 2012; Sarac et al., 2013) or lower (Do et al., 2013; Lisi et al., 2014) limb exoskeleton robots (Lisi and Morimoto, 2017).

Most notably, clinical trials have been carried out to verify the effectiveness of ERD-based brain robot interfaces (BRI) for motor function recovery (Ang et al., 2009, 2014; Broetz et al., 2010; Caria et al., 2011; Shindo et al., 2011; Ramos-Murguialday et al., 2013; Naros and Gharabaghi, 2015). In these works, the motion of a neuroprosthesis is either triggered (Shindo et al., 2011) or continuously controlled (Ramos-Murguialday et al., 2012) based on the output of a BCI. In this context, minimizing the latency between the motor intention onset and the device activation is critical (Muralidharan et al., 2011), since it has been hypothesized that simultaneous contingent association between brain oscillations and proprioceptive feedback leads to superior associative learning and elicits motor learning (Ramos-Murguialday et al., 2012, 2013). For the same reason, an asynchronous BCI strategy, where a decoder continuously estimates the mental state (i.e., rest vs. motor imagery) of a subject, is more suitable. Here asynchronous refers to the fact that a decoder continuously analyses the EEG data, irrespectively of the cue given to the subject, trying to maximize true positives during the motor imagery and to minimize the false positives during the rest or idling state (Townsend et al., 2004).

**Figure 1:**
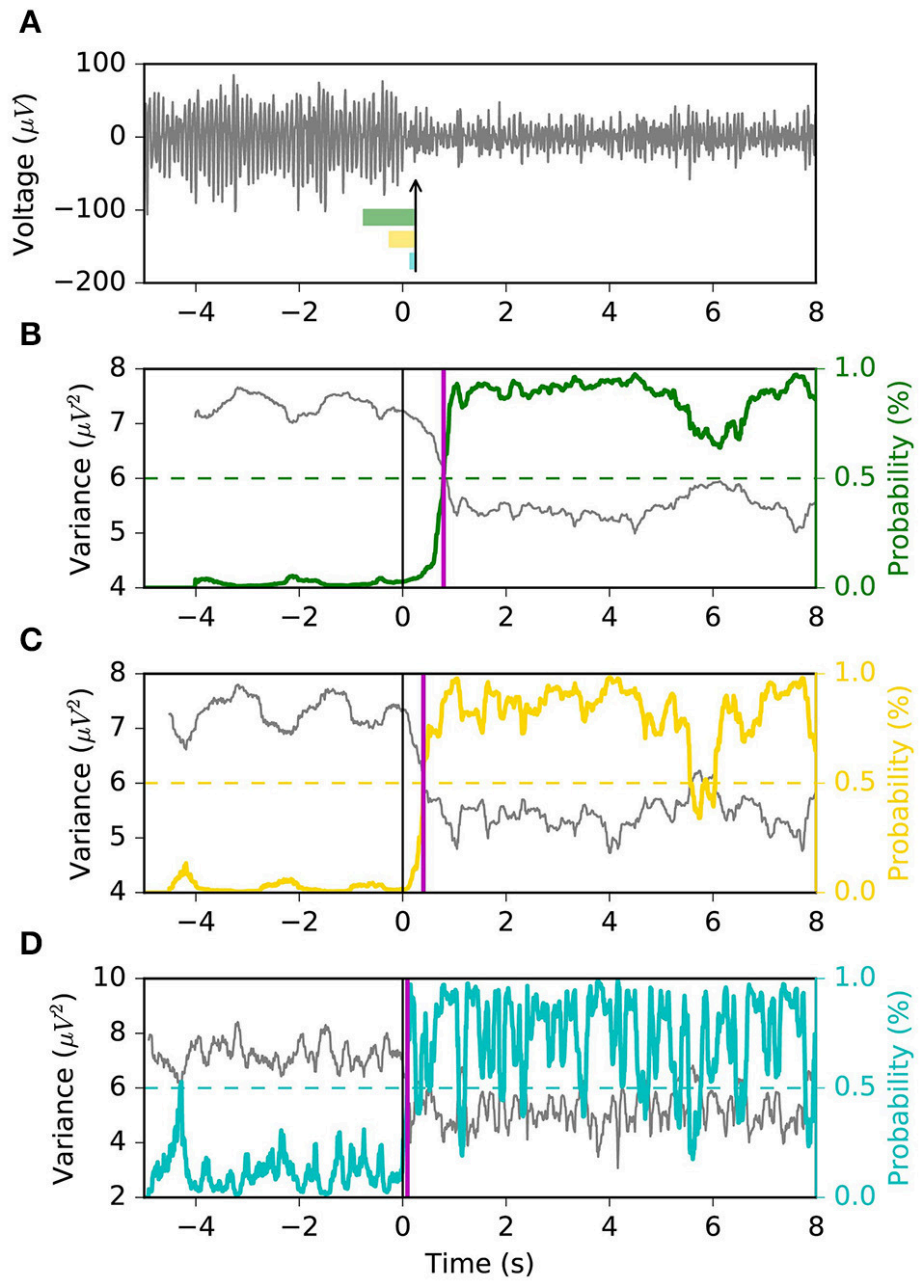
Problem statement. The sliding window approach introduces a delay in the ERD detection. A long sliding window achieves a stable output (i.e., less false positives and more true positives) at the cost of a larger detection latency. A shorter sliding window is faster at detecting the ERD, but its output is less stable and has larger variability. Indeed, it is important to note that the left vertical axis of **(D)** covers a broader range. Panel **(A)** shows the EEG voltage obtained after optimal bandpass filtering and CSP spatial filtering (using the most discriminative CSP component) as described in section 2.4.3. The three rectangles represent the sliding window lengths (i.e., 1.0, 0.5, and 0.1 s) used for the models shown in **(B–D)**. The vertical arrow indicates the point in time corresponding to the output of the depicted sliding windows. Panels **(B–D)** represent the decoding associated with sliding window lengths 1.0, 0.5, and 0.1 s, respectively. Each shows the variance, computed on the signal in **(A)** by the respective sliding window, with a solid gray line. The probability output of the logistic regression classifier is drawn with a solid colored line. The vertical solid black line shows the time of the motor imagery cue onset. The horizontal dashed colored line shows the classifier's threshold with respect to the classifier's probability output (solid colored line). The vertical solid magenta line shows the estimated latency of detection. This example is taken from a subject performing left motor imagery.

Asynchronous BCI has received less attention compared to synchronous BCI (Lotte et al., 2007). The most conventional approach to real-time SMR-based asynchronous decoding is sliding window classification (Townsend et al., 2004; Muralidharan et al., 2011; Shindo et al., 2011; Ang et al., 2012; Lisi et al., 2016). In such systems, a sliding window is required in order to compute statistics, e.g., variance, associated with the spectral features of the signal. Usually, longer sliding windows (i.e., 1 s) are chosen since they provide smooth variance estimates over time and a more stable output, which comes at the cost of a larger latency in ERD detection. Reducing the threshold of the classifier may reduce the latency, at the cost of a larger false positive rate (Muralidharan et al., 2011). On the other hand, shorter windows would minimize the latency, with the drawback of a higher feature variability and unstable output. Therefore, it becomes clear (Figure 1) that such systems are characterized by a trade-off between detection latency, false positive rate (FPR) and true positive rate (TPR).

**Figure 2:**
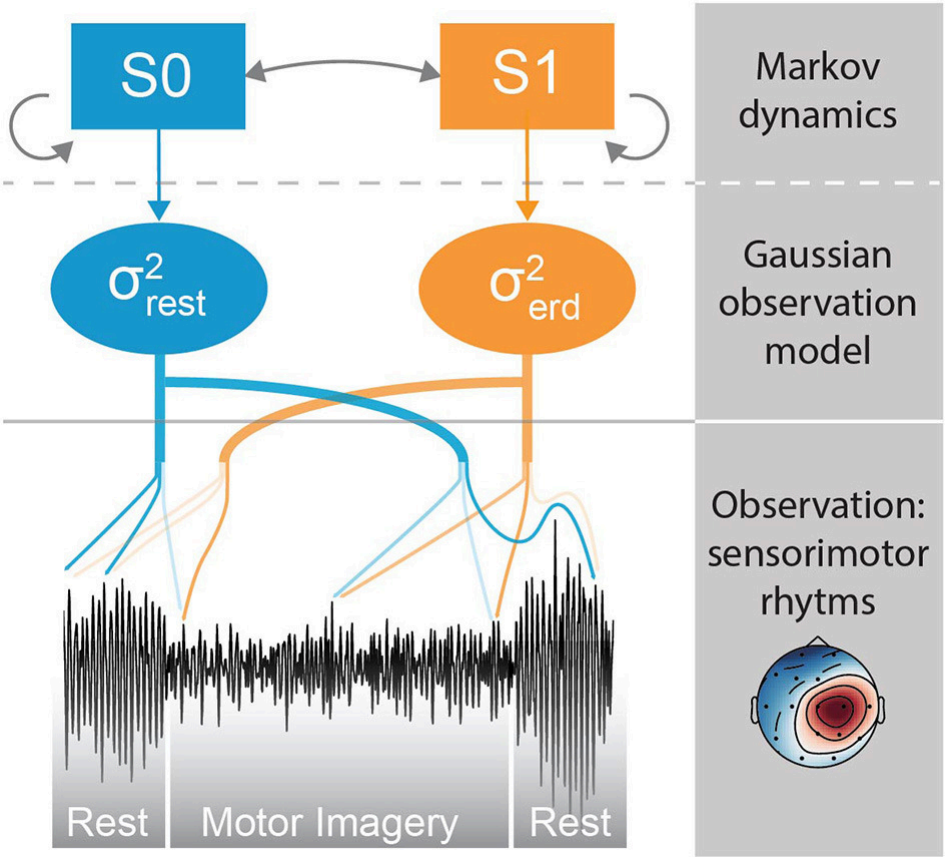
Conceptual representation of the Markov switching model (MSM). The sensorimotor rhythms are obtained by CSP spatial filtering. The MSM is composed of a Gaussian observation model whose variance is governed by Markovian dynamics. If the variance of the sensorimotor rhythm is high, the probability of state *S*0 is high; if the variance is low, the probability of *S*1 is high. The probability of *S*1 represents the probability of an ERD.

Here, we propose to avoid the use of a sliding window for the explicit calculation of the signal variance, and instead estimate the likelihood of the ERD state directly from the instantaneous EEG voltage. Based on the observation that often times the ERD occurs abruptly, we use a Markov switching model (MSM), a method that is suitable in applications where the latent state of a system changes suddenly, such as in the economics field (Hamilton, 2010). In our implementation, the model takes the form of a Gaussian observation model whose variance is governed by two latent discrete states with ergodic (i.e., fully connected) Markovian dynamics. The objective is to estimate the brain state (i.e., rest vs. ERD) given the EEG voltage, spatially filtered by common spatial pattern (CSP), as observation (Figure 2). The two variances associated with the two latent states are calibrated offline using the variance of the CSP projection during rest and motor imagery, respectively. Intuitively, the first state (*S*0) has larger probability during the baseline resting period, while the second state (*S*1) has larger probability during motor imagery. The transition matrix of the latent states is optimized by using a variant of the “quickest detection” strategy (Poor and Hadjiliadis, 2009) that minimizes a cost function of detection latency and false positive rate. A similar approach has been taken in the development of an online seizure detection method based on intracranial EEG (Santaniello et al., 2011).

Markov and state space models in general, are not widespread within the non-invasive BCI community, but they are promising classifiers (or decoders) for BCI systems (Lotte et al., 2007). In literature, Hidden Markov models (HMM) have been proposed with the objective of maximizing classification performance of left vs. right motor imagery, without explicitly targeting the rest class nor the trade-off between detection latency and FPR reduction. The HMM proposed in previous works (Obermaier et al., 2001; Chiappa and Bengio, 2003; Cincotti et al., 2003; Rezaei et al., 2006) are left-to-right finite automata meant to model the temporal changes of the EEG during a motor imagery task. The rationale is that sensorimotor rhythms have specific temporal characteristics, e.g., a short-latency ERD is often followed by a ERS. Each class of interest is assigned a separate HMM trained with trials from that specific class. An unknown trial is classified according to the HMM model with the highest probability, as calculated by Viterbi algorithm. Therefore, each HMM represents one class, and each state of a HMM represents a specific temporal state of that class. In Obermaier et al. (2001) and Rezaei et al. (2006) the classification is done synchronously, meaning that the rest class is not modeled. In Cincotti et al. (2003) and Chiappa and Bengio (2003) the classification is asynchronous, however the rest class is not modeled and not evaluated (i.e., FPR not computed). The observations of an HMM are spectral (Obermaier et al., 2001; Cincotti et al., 2003) or statistical features (Rezaei et al., 2006) of the EEG signal computed on a sliding window. Such models are not suitable to classify rest vs. motor imagery, since temporal dynamics during rest are not well determined. Moreover, the need of a sliding window for feature extraction makes them comparable to a sliding window classifier from a detection latency point of view. On the other hand, the proposed MSM is an ergodic model where no specific temporal sequence is modeled, and any state can be visited at any time. In the MSM the rest class is modeled as a state with high variance, and is included in the evaluation criteria (i.e., FPR). Moreover, a sliding window is not necessary, as previously explained, making it possible to track closely abrupt changes associated with the ERD.

## 2. Methods

**Figure 3:**
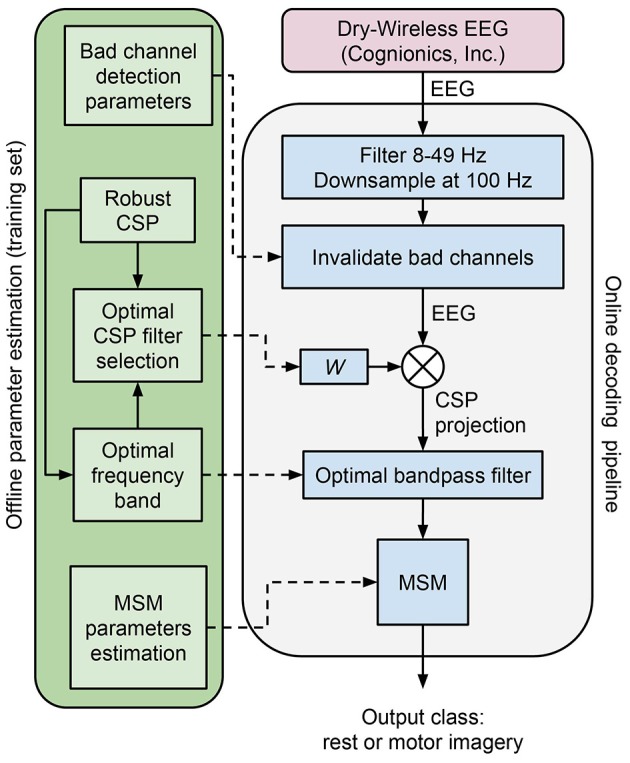
Decoding pipeline. The green box on the left side represents the steps during offline parameter estimation executed on the training set. The blue box on the right is the online decoding pipeline, that uses the parameters estimated offline.

In this section we describe the modules composing the neural decoding pipeline (Figure 3). We begin by explaining data acquisition, followed by online decoding pipeline, the MSM and offline parameter estimation. The last subsection describes the methodology used to assess the performance of the proposed method.

### 2.1. Data acquisition

To explore the MSM model and its application to non-invasive neural decoding, we have carried out motor imagery experiments with 50 healthy subjects: age 24 ± 3, 9 females and 41 males. Only one subject was familiar with motor imagery BCI, while all the others had never performed a motor imagery BCI task before. During a single run, a subject performed 10 trials of cued motor imagery, interleaved by a period of rest. For each subject, 4 runs were collected, interleaved by a few minutes of rest. It should be noted that even though the experiment is cued, the subsequent decoding is done irrespectively of the cue (i.e., asynchronous decoding), and that a cued experiment is needed in order to know the ground truth onset of the motor imagery. For a given subject, the motor imagery type was fixed. The majority of the participants (*N* = 47) was randomly assigned either to a left (*N* = 25) or right (*N* = 22) hand motor imagery task, with 7 s rest duration and 5 s motor imagery duration. The additional 3 subjects performed a foot motor imagery task with 8 s rest duration and 4 s motor imagery duration. The EEG signal was collected at a sampling rate of 500 Hz, by the Quick-20 dry-wireless headset (Cognionics, Inc.), which is a full 10–20 array, with 19 channels (F7, Fp1, Fp2, F8, F3, Fz, F4, C3, Cz, P8, P7, Pz, P4, T3, P3, O1, O2, C4, T4) plus reference on A1 and ground on A2. Participants gave written informed consent for the experimental procedures, which were approved by the ATR Human Subject Review Committee.

### 2.2. Online decoding pipeline

Here we describe the online decoding pipeline (Figure 3, blue box on the right), that uses the optimal parameters computed offline by the method detailed in section 2.4. A subset of channels that is typically associated with sensorimotor activity is selected (i.e., F3, Fz, F4, C3, Cz, Pz, P4, P3, C4). The signal is bandpass filtered in the 8–49 Hz range and downsampled at 100 Hz. Outlying channels are identified using the deviation criterion (see section 2.4.1), and temporarily invalidated by assigning them the average of the valid channels. Subsequently, the projection of the most discriminative CSP filter is computed and further bandpass filtered within the optimal frequency band. The bandpass filtered CSP projection is then given as an observation to the MSM in order to estimate the current class (i.e., rest or motor imagery).

### 2.3. Markov switching model (MSM)

The MSM was first introduced in the field of econometrics (Hamilton, 1989; Turner et al., 1989), but to the authors' knowledge it has never been applied on EEG motor imagery data. The model is sketched in Figure 2, and it consists of a Gaussian observation model whose variance is governed by two latent states with Markovian state transitions. The observation model is a simple zero-mean Gaussian with variance *σ*^2^:

(1) $$\begin{array}{l} y_{t}\sim N(0,\sigma_{t}^{2}) \end{array}$$

where *y*_*t*_ is the observation at time *t* and $\sigma_{t}^{2}$ is dependent on a discrete state *S*_*t*_:

(2) $$\begin{array}{l} \sigma_{t}^{2}=\{\begin{array}{ll} \sigma_{rest}^{2} & {\text{if }S}_{\text{t}}=\;0\\ \sigma_{erd}^{2} & {\text{if }S}_{t}=1 \end{array} \end{array}$$

where *S*_*t*_ = 0 is associated with the resting state, and *S*_*t*_ = 1 is associated with a motor imagery ERD. Since *S*_*t*_ is hidden (i.e., we do not know when to apply each sub-model), we use a weighted combination of each sub-model (i.e., *soft switching*), where the weights are given by *P*(*S*_*t*_ = *i*|*y*_1 : *t*_). Therefore, the resulting system can be thought of as a mixture of Gaussian models (Murphy, 1998).

The discrete state *S*_*t*_ = 0 or 1 denotes the latent space of the system and it is generated by a realization of a first-order Markov process with transition probabilities defined by the matrix:

(3) $$\begin{array}{l} Z=\left[\begin{array}{cc} p & 1-p\\ 1-q & q \end{array}\right], \end{array}$$

where *p* = *P*(*S*_*t*_ = 0|*S*_*t*_ = 0) and *q* = *P*(*S*_*t*_ = 1|*S*_*t*_ = 1) represent the probability of remaining in *S* = 0 or remaining in *S* = 1, respectively. The offline calibration of the variances $\sigma_{rest}^{2}$, $\sigma_{erd}^{2}$, and probabilities *p* and *q* is described in section 2.4.4.

At each iteration, the new probability of each discrete state is computed according to Bayes' theorem:

(4) $$\begin{array}{l} \text{posterior}=\frac{\text{prior}\times \text{likelihood}}{\text{normalization factor}} \end{array}$$

The *likelihood* for each discrete state *i* is:

(5) $$\begin{array}{l} L_{i}=\frac{1}{\sqrt{2\pi \sigma_{i}^{2}}}{\text{e}}^{-y^{2}/2\sigma_{i}^{2}} \end{array}$$

where $\sigma_{i}^{2}$ is either $\sigma_{rest}^{2}$ or $\sigma_{erd}^{2}$ and *y* is the EEG voltage spatially filtered by a CSP filter (see section 2.4.2). The *prior* in Equation 4, which we denote by $\bar{\text{c}}$, is computed by propagating the previous probabilities of the discrete states according to the transition probability matrix ***Z***:

(6) $$\begin{array}{l} \bar{c}=Z.\mu \end{array}$$

where μ is the vector containing the previous probabilities of the discrete states. The numerator of Equation 4 can simply be computed as:

(7) $$\begin{array}{l} {\tilde{\mu }}_{i}={\bar{c}}_{i}\times L_{i} \end{array}$$

and then normalized (i.e., *normalization factor*) so that it sums to one:

(8) $$\mu_{i}=\frac{{\tilde{\mu }}_{i}}{\sum_{j=0}^{1}{\tilde{\mu }}_{j}}$$

The new probability of each discrete state is contained in *μ*_*i*_.

Given a CSP projection observation *y*_*t*_ at time *t*, the probability of state *S*_*t*_ = 0 associated with variance at rest ($\sigma_{rest}^{2}$) is larger when the subject is in resting state, while the probability of state *S*_*t*_ = 1 associated with variance during motor imagery ($\sigma_{erd}^{2}$) is larger when the subject does motor imagery.

### 2.4. Offline parameters estimation

Offline parameter estimation (Figure 3, green box on the left) is performed on the training data set, before running online decoding. Signal preprocessing is equivalent to the one used for online decoding.

#### 2.4.1. Bad channel detection parameters

We implemented two types of bad channel detection methods, based on the *correlation* and *deviation criterions*, respectively (Bigdely-Shamlo et al., 2015). The former criterion labels a channel as bad if its maximum correlation coefficient with the other channels is below a threshold (0.4 by default). Here, the correlation coefficients are computed on the same epochs used by the CSP algorithm (section 2.4.2). This procedure is carried out offline and, once a channel is rejected by the correlation criterion, it is removed completely from both offline parameters estimation and online decoding. On the other hand, the *deviation criterion* is applied at each time sample of both offline and online processing. In the deviation criterion, outlying channels are identified using the modified Z-score method (Iglewicz and Hoaglin, 1993): a channel *i* at time *t* whose modified Z-score $M_{i}\left(t\right)=0.6745\left(x_{i}\left(t\right)-\bar{x_{i}}\right)/MAD_{i}$ is larger than 5 is considered as an outlier. The parameters $\bar{x_{i}}$ and *MAD*_*i*_ are computed using the entire EEG of the training set and they represent the median and the median absolute value (MAD) of the EEG channel, respectively. Channels marked as bad at time *t* are temporarily invalidated by assigning them the average of the valid channels. In the case that most channels are bad, the minority would still be used online to compute final decoding and evaluate the performance measures.

#### 2.4.2. Robust CSP

**Figure 4:**
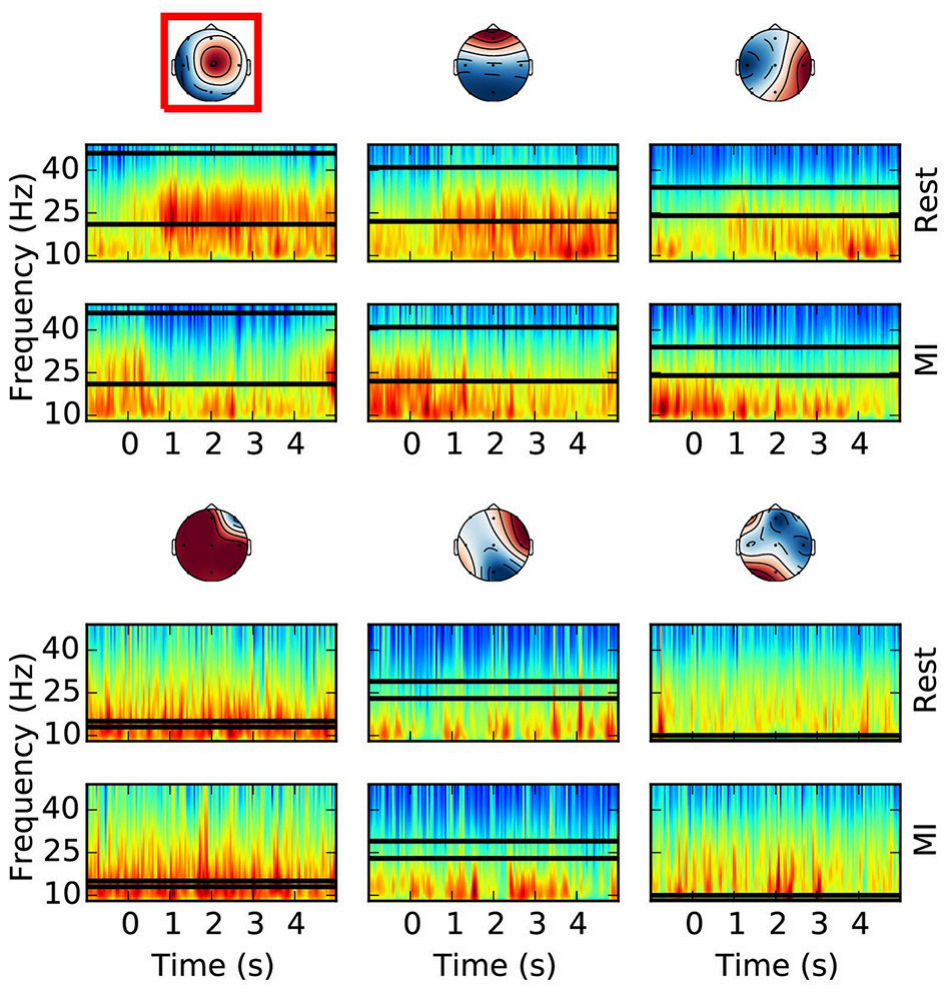
Offline optimal frequency band and CSP filter selection. For each CSP filter the spectrograms of the rest **(top)** and MI **(bottom)** conditions are shown. The selected optimal frequency band is depicted with two horizontal lines over the time frequency representation. The optimal CSP filter, selected among the first 3 and last 3 columns of the unmixing matrix, is surrounded by a red box. The color scale of the time-frequency representation is equalized for each column (i.e., CSP), and for the selected CSP it is between 5.4 and 8.9 with unit $10log_{10}\left(\mu V^{2}/Hz\right)$. All the other CSPs have comparable color ranges. This example is taken from a subject performing foot motor imagery. It is possible to appreciate how the optimal frequency band and CSP filter contain the most discriminative (i.e., rest vs. MI) spectral features.

A robust version of the CSP algorithm (Yong et al., 2008) is executed on the training set to estimate the spatial filters, robust to outliers, that maximize the difference in variance between the two classes (i.e., rest vs. motor imagery). For this purpose, from the bandpass filtered EEG signal (8–49 Hz) of the training set, we cut epochs of 2 s length from 1 s to 3 s with respect to the motor imagery onset, for the motor imagery class. For the longer rest class, the epochs start 1 s after rest onset and end 1 s before rest offset. The length discrepancy between motor imagery and rest epochs is due to the fact that we want the covariance matrix of the rest class to capture as much as possible the variability of the rest EEG, to make the CSP filters more robust. Finally, we select the first 3 and last 3 columns of the CSP unmixing matrix, representing the most discriminative spatial filters (Figure 4, top row). The CSP filters selected at this point are redundant, so that the CSP filter selection at the next step has enough options to choose from.

#### 2.4.3. Optimal frequency band and CSP filter selection

For each CSP filter, the most discriminative frequency band (Figure 4) is computed using the heuristic proposed in Blankertz et al. (2008). Frequency bands with a high correlation coefficient between the logpower and classes labels are iteratively added to the optimal frequency band: the frequency with the largest correlation is selected and then the adjacent frequencies (i.e. above and below) are added if their correlation is at least 90% of the best correlation. Correlation coefficients are computed on the spectrogram of the same epochs used for CSP (section 2.4.2). Likewise, in order to select the most discriminative CSP filter (Figure 4), we compute the average logpower within the optimal frequency band of each filter, and keep the one having the largest correlation coefficient with classes labels.

#### 2.4.4. MSM parameters estimation

The parameters $\sigma_{rest}^{2}$ and $\sigma_{erd}^{2}$ are assigned the variances of the CSP projection during rest and motor imagery, respectively. Contrary to the CSP epochs, the durations of the rest (i.e., [−3 *s*, −1 *s*] with respect to onset) and motor imagery (i.e., [1 *s*, 3 *s*] with respect to onset) time epochs are chosen to be the same, so that the chance of them containing outliers is minimized. Indeed, it is important to remove epochs with extreme values, in order to obtain an unbiased estimate of variance. Therefore, variance is computed for all the epochs; then, epochs whose variance exceeds 3 standard deviations from the mean are removed. Once data is clean, the average variance of the rest and motor imagery epochs are computed.

For convenience sake, the transition probabilities (*p, q*) of the latent states are defined using the exponential duration model $\pi_{d}=1-\frac{1}{d}$, where *d* is the duration in number of frames and π_*d*_ is the probability of staying in a given state Rabiner (1989). Then, according to a modified “quickest detection” strategy (Poor and Hadjiliadis, 2009; Santaniello et al., 2011), the tuple (*p, q*) is optimized by minimizing a cost function of detection latency (δ*t*) and false positive rate (FPR):

(9) $$\begin{array}{l} C=\delta t+FPR \end{array}$$

The optimization is carried out by Sequential Least Squares Programming (Kraft, 1988), and the starting point of the optimization is set to the probabilities (*p, q*) computed from the durations of rest and motor imagery, respectively. With respect to the cost function, *FPR* is the false positive rate computed using the samples during the *rest* condition. Computing the detection latency δ*t* is more complex, since it is important to avoid irrelevant short-lasting false positives. For this purpose, we use a sliding window approach to find the most robust state transition *S*0 → *S*1 across a trial. Accordingly, the detection time is the *t*_*d*_ that maximizes the difference ${\bar{S}}_{post}-{\bar{S}}_{pre}$, where ${\bar{S}}_{post}$ is the average estimated state *S* in the range [*t*_*d*_, *t*_*d*_ + 3 *s*] and ${\bar{S}}_{pre}$ is the average state *S* in the range [*t*_*d*_ − 1 *s, t*_*d*_]. The rationale for having a longer range for ${\bar{S}}_{post}$ is that in case of several *S*0 → *S*1 transitions, the one with the longer stay in *S*1 should be considered as the real ERD. Once *t*_*d*_ is computed, δ*t* = *t*_*d*_ − *t*_*o*_, where *t*_*o*_ is the time of the motor imagery cue onset. Trials that cannot be improved (i.e., FPR = 0) or trials where ERD does not exist (TPR = 0) should be excluded from the optimization. Based on this rationale and in order to reduce computational cost, only the five most improvable trials within the training set are used for optimization. This is done by looking for the trials in the training set that jointly minimize the absolute difference between FPR and the average FPR (i.e., $\left|FPR-\bar{FPR}\right|$) and maximize TPR.

### 2.5. Performance evaluation

Leave-one-run-out cross-validation is used, separately for each subject, to evaluate the online performance of the proposed decoder: *n* − 1 runs are used as training set and 1 run as test set, so that every run is used as test set once.

#### 2.5.1. Baseline models

The MSM model is compared against a sliding window logistic regression (LR) classifier. Specifically, three different window's lengths are used: 1.0 s, 0.5 s, 0.1 s (i.e., *LR* 1.0 *s*, *LR* 0.5 *s*, *LR* 0.1 *s*). The sliding window includes time samples prior to the current time, as shown in Figure 1, in order to avoid using future information. A logistic regression classifier is trained and tested on the log-transformed sliding window variance of the optimal CSP projection (Townsend et al., 2004). The decoding pipeline up to classification is the same as the one used for MSM. During classifiers' training, the time ranges used to represent the *rest* and *motor imagery* classes are equal to the ones used to tune the MSM: the sliding window whose last sample is within [−3 *s*, −1 *s*] and [1 *s*, 3 *s*], with respect to motor imagery onset, is assigned to the *rest* and *motor imagery* class, respectively. During online decoding and performance evaluation, the positive class of the classifier (i.e., motor imagery) corresponds to *S*1 of the MSM.

#### 2.5.2. Online performance measures

Three measures are used within each trial of each subject for model evaluation: the latency of detection (δ_*t*_), the false positive rate (FPR), and the geometric mean (Barandela et al., 2003) of true positive rate (TPR) and true negative rate (TNR).

The latency of detection δ_*t*_ has beed described in section 2.4.4. Here, however, we need to find consistent δ_*t*_ values across the four models being compared (i.e., MSM, *LR* 1.0 *s*, *LR* 0.5 *s*, *LR* 0.1 *s*), and make sure that state estimates instability would not lead to incompatible δ_*t*_ values across models. Indeed, noisy state estimates of *LR* 0.5 *s* and *LR* 0.1 *s* may have a maximum of the value ${\bar{S}}_{post}-{\bar{S}}_{pre}$ at a very different δ_*t*_ compared to the more stable MSM and *LR* 1.0 *s*, making the comparison difficult and often unfair. Such noisy state estimates would already be accounted for by the TPR and FPR. Therefore, we decided no to judge them again from the latency point of view, and force their latency to be close to that of the more stable models. We do this by extending the procedure described in section 2.4.4: first we find a common point of reference Δ_*t*_ between the more stable models MSM and *LR* 1.0 *s*, then we compute the δ_*t*_ of each model with respect to Δ_*t*_. The common point of reference Δ_*t*_ is found by applying the sliding window procedure (described in section 2.4.4) over the average estimated class of MSM and *LR* 1.0 *s*. Then, for each model we look for the time *t*_*d*_ closest to Δ_*t*_, where a transition *S*0 → *S*1 occurs. Latency is computed as δ*t* = *t*_*d*_ − *t*_*o*_, where *t*_*o*_ is the time of the motor imagery cue onset.

The FPR, TPR, TNR and G-mean are computed for each trial (i.e., consecutive rest and motor imagery) assuming that samples up to 4 s before motor imagery cue onset belong to the negative class, and samples during the motor imagery cue belong to the positive class. The geometric mean ($\text{G-mean}=\sqrt{TPR\cdot TNR}$) is used here as an estimator of accuracy separately for each trial. It should be noted that if in a given trial there is no ERD, even an optimal decoder would produce an uninformative output: correct classification only 50% of the time samples during rest (i.e., *TNR* = 0.5) and motor imagery (i.e., *TPR* = 0.5), yielding G-mean = 0.5. Conversely, for a trial containing an ERD, a sufficiently good decoder is expected to have at least *TNR* = 0.6 and *TPR* = 0.6, yielding G-mean = 0.6. One attractive property of G-mean (Barandela et al., 2003) is that it penalizes unbalanced TPR and TNR, compared to the arithmetic mean, e.g., $\sqrt{0.8\cdot 0.4}<\sqrt{0.6\cdot 0.6}$, (0.8 + 0.4)/2 = (0.6 + 0.6)/2. G-mean and FPR measure the ability of a decoder of estimating the correct state at each point in time; therefore, they are indicative of the decoder output stability (Figure 1).

Models are evaluated on all the trials from all the subjects according to their TPR and FPR (see Table 1), in order to verify that the baseline classification performance is in agreement with previous studies of asynchronous BCI, and to verify that decoding performance is similar across motor imagery tasks (i.e., foot, left and right hand motor imagery).

**Table 1:** Mean and standard deviation, of true positive rate (TPR) and false positive rate (FPR) of each model, computed across across 50 subjects and including all the trials, i.e., before removing trials with no ERD.

	**MSM**	**LR1.0**	**LR0.5**	**LR0.1**
TPR	0.64 ± 0.14	0.62 ± 0.11	0.60 ± 0.10	0.55 ± 0.08
FPR	0.37 ± 0.11	0.37 ± 0.10	0.38 ± 0.08	0.41 ± 0.06

Not every subject is able to produce an ERD at every trial, due to the so called BCI illiteracy (Vidaurre and Blankertz, 2010) and to the fact that sensorimotor rhythms are noisy signals. This means that several trials do not contain an ERD at all. In such cases, it is uninformative to compare different models, since all will fail due to the absence of an ERD. Therefore, to obtain a meaningful comparison across models, it is necessary to remove such trials. Here, a trial with no ERD is defined as a trial where neither the MSM nor the best sliding window classifier (i.e., *LR* 1.0 *s*) are able to produce a sufficiently accurate and stable output. Based on this assumption, a trial with a G-mean smaller than 0.6 is considered as a trial with no ERD. This threshold is a compromise between chance level (i.e., 0.5) and the threshold commonly accepted in BCI (i.e., 0.7) as the minimum needed for communication (Nijboer et al., 2008). Subjects who could not produce an ERD in at least 25% of the trials are removed from the analysis. This threshold was chosen based on the fact that one trial contains two classes. Therefore, the probability of a successful trial by chance is 25%: the combination of *majority of true negatives (TN)* and *majority of true positives (TP)* against all the other 3 combinations (e.g., *majority of false negatives (FN)* and *majority of TN*). Based on this approach, 2 out of 50 subjects were removed. The percentage of successful trials for the retained subjects was on average 58 ± 18 (SD) (chance level at 25%).

#### 2.5.3. Statistical testing

For each of the three performance measures (i.e., latency, FPR and G-mean) we compare MSM with the sliding window classifiers (i.e., *LR* 1.0 *s*, *LR* 0.5 *s*, *LR*0.1*s*) by Analysis of variance (ANOVA) with a linear mixed-effects model (Bates et al., 2015), specified by the formula, in Wilkinson notation:

$$\begin{array}{l} \text{Measure}=\text{Decoder}+(1+\text{Decoder}|\text{Subject})+\\ \;(1|\text{Subject}:\text{Trial}) \end{array}$$

For each measure (i.e., latency, FPR or G-mean), one new model is inferred. Therefore, the dependent variable Measure is either latency, FPR or G-mean. The fixed-effect predictor Decoder represents the type of decoder (i.e., MSM, *LR* 1.0 *s*, *LR* 0.5 *s*, *LR* 0.1 *s*). The term (1+Decoder|Subject) represents a random intercept and random slope for Decoder, which allows for different random relationships among the decoders for each subject. The term (1|Subject:Trial) allows for different random intercepts for each trial of each subject. Tukey's all-pair comparisons *post-hoc* tests are carried out by *z*-test, corrected for multiple comparisons by the Holm-Bonferroni procedure (Bretz et al., 2010).

## 3. Results

The true positive rate (TPR) and false positive rate (FPR) of the models before removing trials with no ERD are shown in Table 1. This allows us to verify whether the baseline classification performance is in agreement with previous studies of asynchronous BCI. The performance measures of MSM and *LR* 1.0 *s* are approximately 10% lower than the results reported in Townsend et al. (2004) in which 3 healthy subjects, achieved on average an FPR of 0.27 and a TPR of 0.74. This may be due to the fact that while the 3 subjects of Townsend et al. (2004) were all familiar with the BCI, in our experiment 49 out of 50 subjects had never experienced a motor imagery BCI task. Thus, a consistent portion of these naive subjects may have been labeled as BCI illiterate (Vidaurre and Blankertz, 2010). Indeed, our averaged performance is in line with the least performing subject in Townsend et al. (2004). Moreover, before removing trials with no ERD, we verify that MSM decoding performance is similar across motor imagery tasks (i.e., foot, left and right hand motor imagery) as measured by G-mean: 0.66 ± 0.07 for foot, 0.61 ± 0.08 for left hand and 0.64 ± 0.07 for right hand. Such similarity across tasks is confirmed, for every decoder, by the large *p*-values (*p* > 0.1) of a one-way ANOVA with G-mean as response variable and motor imagery tasks as groups.

The linear mixed-effects ANOVA tests, computed on the trials with ERD, showed that the effect of the type of decoder (i.e., MSM, *LR* 1.0 *s*, *LR*0.5*s*, *LR* 0.1 *s*) was significant on latency [*F*_(3)_ = 79.09, *P* < 2.2 × 10^−16^], on FPR [*F*_(3)_ = 92.99, *P* < 2.2 × 10^−16^] and on G-mean [*F*_(3)_ = 212.36, *P* < 2.2 × 10^−16^]. The magnitude and significance of all the pairwise comparisons are shown in Table 2.

**Table 2:** Result of multiple comparisons.

	**Latency**		
	**Estimate**	***z*-value**	**Pr(>|z|)**
LR1.0 - MSM	0.155	4.48	2.22 × 10^−5^
LR0.5 - MSM	−0.0908	−2.46	0.014
LR0.1 - MSM	−0.152	−5.29	4.96 × 10^−7^
LR0.5 - LR1.0	−0.246	−15.1	0
LR0.1 - LR1.0	−0.307	−12.6	0
LR0.1 - LR0.5	−0.0616	−3.63	0.000561
	**False positive rate (FPR)**		
	**Estimate**	***z*****-value**	**Pr(**>|**z**|**)**
LR1.0 - MSM	0.0011	0.0726	0.942
LR0.5 - MSM	0.0419	2.78	0.0108
LR0.1 - MSM	0.0984	6.43	3.79 × 10^−10^
LR0.5 - LR1.0	0.0408	11.1	0
LR0.1 - LR1.0	0.0973	16.3	0
LR0.1 - LR0.5	0.0565	11.5	0
	**G-mean(TPR,TNR)**		
	**Estimate**	***z*****-value**	**Pr(**>|**z**|**)**
LR1.0 - MSM	−0.0144	−1.77	0.0774
LR0.5 - MSM	−0.0508	−5.88	8.21 × 10^−9^
LR0.1 - MSM	−0.11	−13.7	0
LR0.5 - LR1.0	−0.0365	−12.1	0
LR0.1 - LR1.0	−0.0958	−23.4	0
LR0.1 - LR0.5	−0.0594	−17.7	0

*Each subtable contains the results of latency, FPR and G-mean, respectively. Each row contains one pairwise comparison between two models. The column Estimate contains the estimated average difference between the two models being compared. P-values below 2 × 10^−16^ are assigned P = 0*.

**Figure 5:**
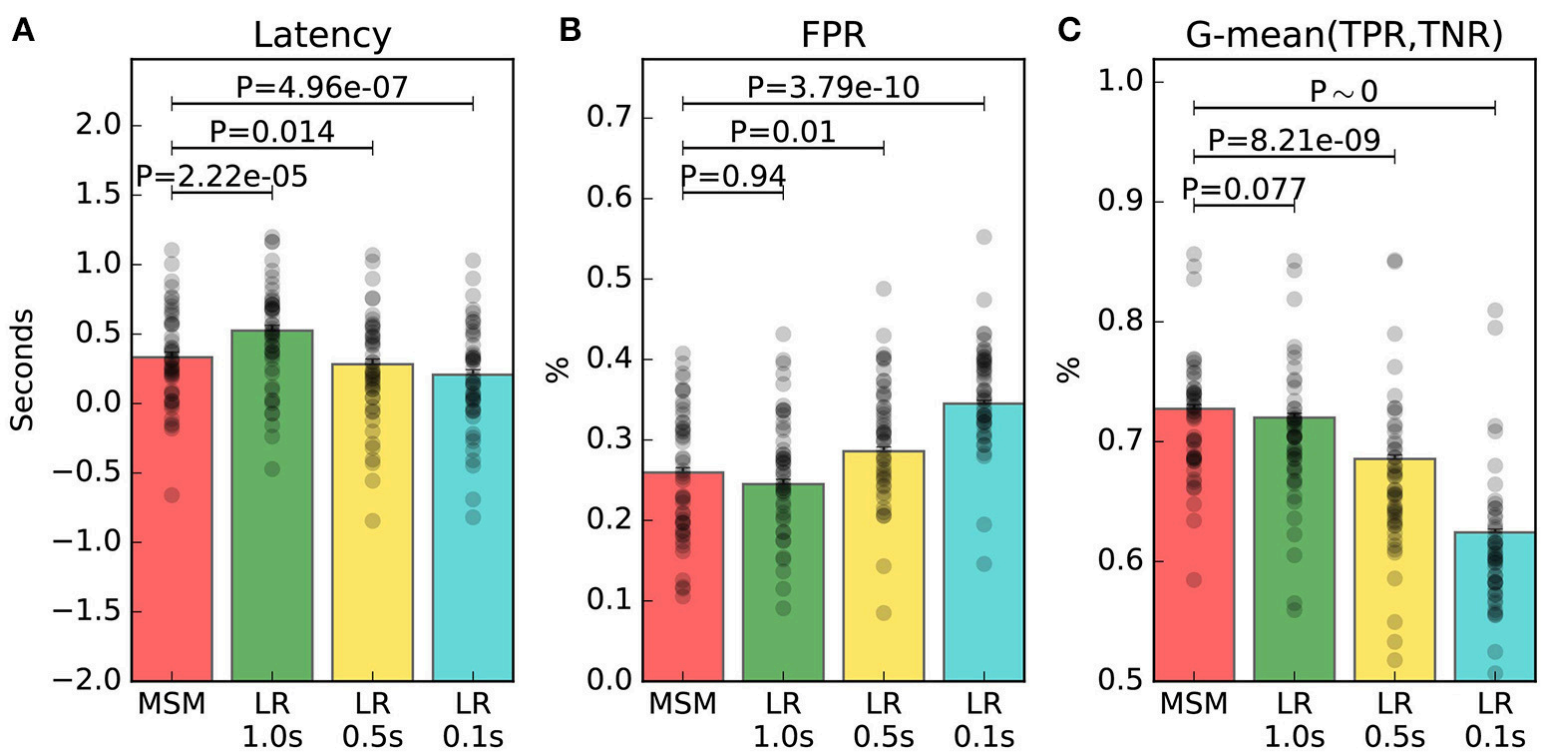
Online performance measures of trials containing an ERD. The average measure of each individual is represented by a black dot. Panel **(A)** shows the latency of detection. Panel **(B)** shows the false positive rate (FPR). Panel **(C)** shows the geometric mean (G-mean) between true positive rate (TPR) and true negative rate (TNR). The significance of the multiple comparisons involving MSM are illustrated, while all the other comparisons are shown in Table 2.

The main result is that MSM achieves the best tradeoff between latency, FPR and G-mean, as depicted in Figure 5. The latency of MSM is on average 155 ms shorter than that of *LR* 1.0 *s*, with significance at *P* = 2.2 × 10^−5^; while FPR and G-mean are not significantly different. Indeed, the estimated averages show that MSM improves G-mean of only 1.4% with significance at *P* = 0.077, and increases FPR of only 0.1% with significance at *P* = 0.94. Moreover, *LR*0.5*s* and *LR* 0.1 *s* achieve significantly better latency but significantly worse FPR and G-mean.

**Figure 6:**
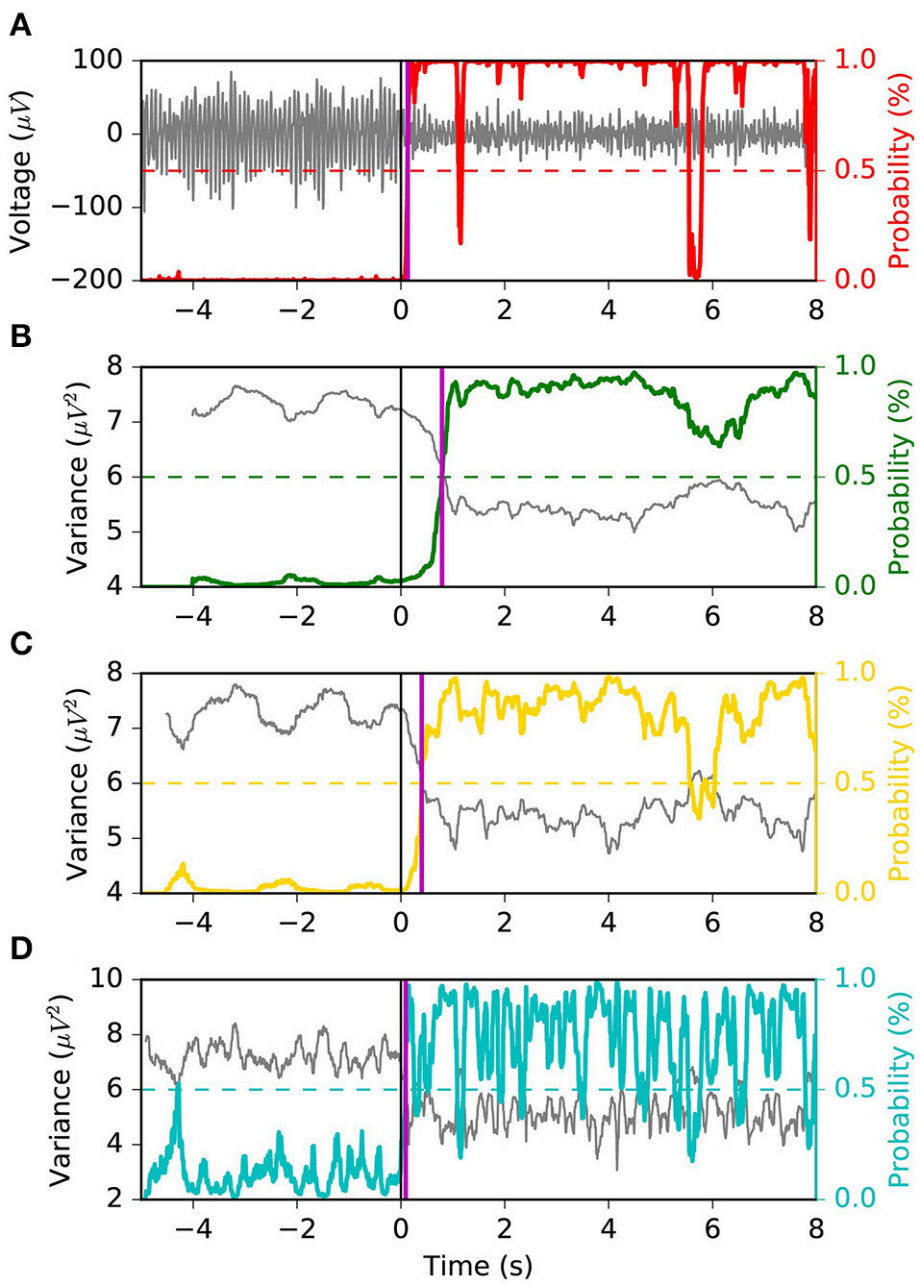
Example 1 of the typical behavior of the four models. The same left motor imagery trial as in Figure 1 is depicted. We observe that the MSM achieves the “quickest detection”, while keeping a stable output. Panel **(A)** shows the EEG voltage obtained after CSP spatial filtering in gray and the MSM model output in red. Panels **(B–D)** represent the decoding associated with sliding window lengths 1.0, 0.5, and 0.1 s, respectively. This figure follows the same convention as in Figure 1.

**Figure 7:**
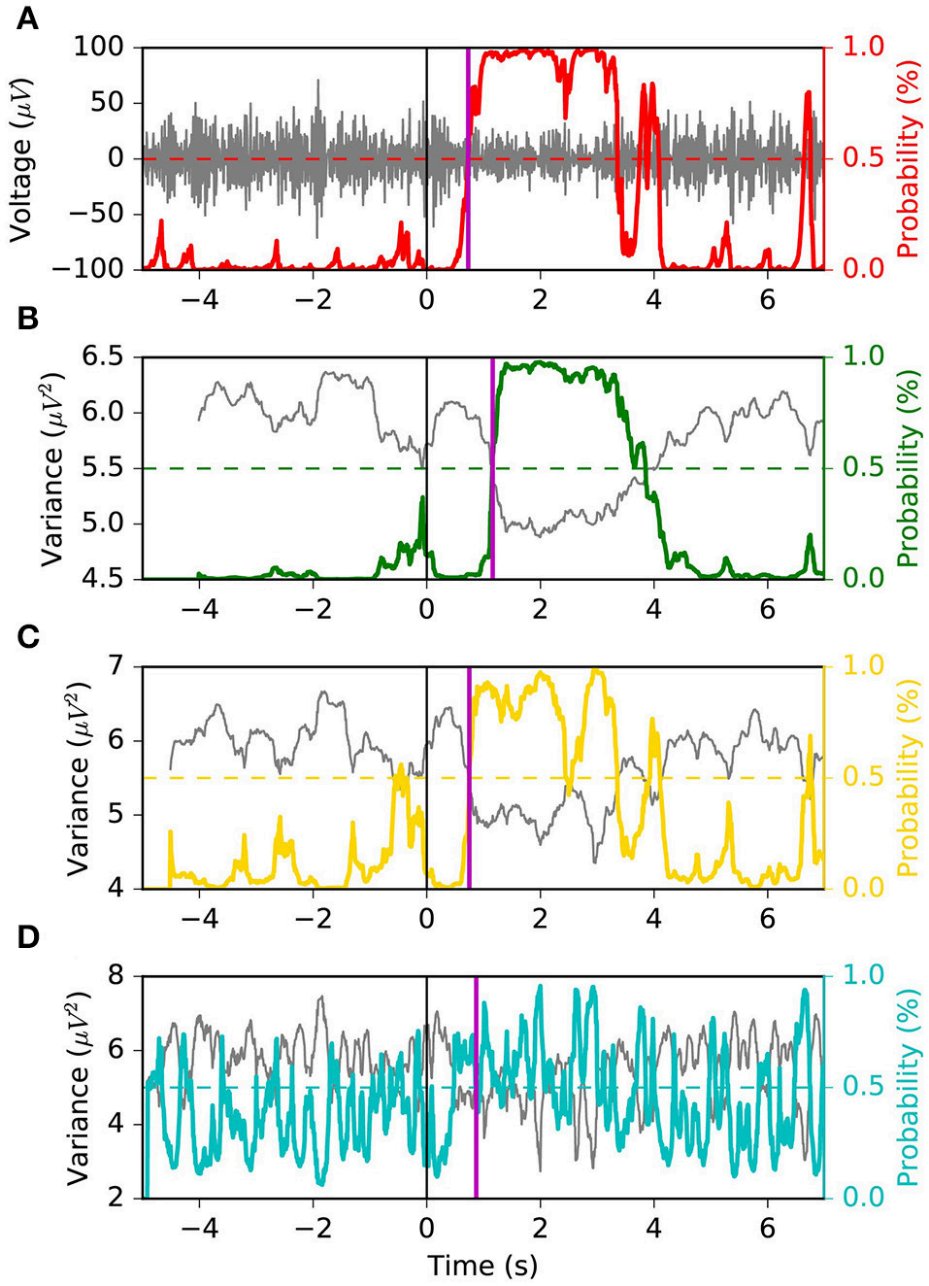
Example 2 of the typical behavior of the four models. This example is taken from a subject performing foot motor imagery. The MSM achieves the best tradeoff between latency, FPR and G-mean. Panel **(A)** shows the EEG voltage obtained after CSP spatial filtering in gray and the MSM model output in red. Panels **(B–D)** represent the decoding associated with sliding window lengths 1.0, 0.5, and 0.1 s, respectively. This figure follows the same convention as in Figure 1.

We appreciate the importance of achieving the best tradeoff between latency, FPR and G-mean, from Figures 6, 7. The former depicts the same left motor imagery trial as in Figure 1, while the latter shows another example from a different subject performing foot motor imagery. In Figure 6 we observe how MSM detects the ERD faster than *LR* 1.0 *s*, *LR*0.5*s* and as quick as *LR* 0.1 *s*, while keeping a stable output. In the example of Figure 7, when comparing MSM with *LR* 1.0 *s* we observe that latency is shorter, FPR is equivalent and G-mean is slightly larger due to more true positives. On the other hand, MSM detects the ERD as quick as *LR*0.5*s*, but with less false positives and more stability. Also, we observe how unstable the output of *LR* 0.1 *s* can be.

## 4. Discussion

We showed that, in asynchronous motor imagery detection, the proposed MSM achieves the best tradeoff between latency of detection, false positive rate and G-mean of true positive rate and true negative rate. This translates into a quicker motor imagery detection (i.e., latency) at no cost of output stability (i.e., FPR and G-mean), compared with sliding window classifiers. On average, the proposed MSM was able to detect an ERD 155 ms faster than the most performant and commonly used sliding window approach (i.e., *LR* 1.0 *s*). The main reason for such improvement is due to the fact that the ERD is a phenomenon that occurs abruptly, and MSM have been historically used in applications where the latent state of a system changes suddenly (Hamilton, 2010).

Given the relatively low signal-to-noise ratio of the EEG signal, the most realistic application of the proposed model is BCI-based neurorehabilitation, where proprioceptive feedback is provided by a neuroprosthesis, based on the patient's brain signal. The control of only one degree of freedom is typically required, and false negatives or positives do not have the potential of becoming disastrous. In this context, the short latency of detection and stable output of the MSM allows for a more reactive proprioceptive feedback and for a reduced delay of contingent feedback. This is a desirable property, since it is hypothesized that simultaneous contingent association between volitionally evoked SMR and proprioceptive feedback may lead to superior associative learning and elicit motor learning (Ramos-Murguialday et al., 2012, 2013).

It should be noted that the ERD does not always occur at the cue onset, but on average it happens a few tenths of a second later. However, the cue onset is the only ground truth available. As a result, the average latencies shown in Figure 5A include the intrinsic delay of ERD occurrence with respect to cue onset. Such delay of ERD occurrence cannot be improved, and should not be taken into account when evaluating the models. Indeed, the relative difference among models' latencies is the most important aspect. In this context, the MSM is able to detect the ERD 155 ms earlier than *LR* 1.0 *s*, meaning that the time required between the ERD occurrence (not necessarily at time 0 s) and the detection is 155 ms shorter, on average. In addition, associative learning is hypothesized to occur with respect to the ERD onset, not the cue onset.

Whether a latency reduction of 155 ms is crucial for improving temporal association, inducing neural plasticity and restoring function is yet to be confirmed. It has been hypothesized that the maximum proprioceptive feedback delay still inducing co-activation in Hebbian plasticity should be 200 or 300 ms, but this remains an open question that needs to be addressed by the community (Grosse-Wentrup et al., 2011; Xu et al., 2015). Muralidharan et al. (2011) discussed that increasing decoding accuracy at the expense of longer latencies (i.e., 200, 400, or 600 ms) would cause delayed neuroprosthetis activation and may limit therapeutic benefits. Another study (Xu et al., 2014) proposed a new method for the detection of movement-related cortical potentials, reporting a 145 ms latency reduction. They discussed that such an improvement is fundamental in order to induce neuroplastic changes in closed-loop BCIs, since the temporal association between movement-related brain signals and the afferent input is crucial for plasticity (Mrachacz-Kersting et al., 2012).

In the neurorehabilitation field of application, a question arises: whether a neurological injury would have impact on the CSP algorithm. We expect the CSP patterns to be different between healthy subjects and patients (Lei et al., 2017). However, previous reports of CSP-based BCI in clinical trials have shown that the algorithm successfully finds the most discriminant spatial filters in stroke patients (Ang et al., 2015). Therefore, retraining the CSP filters individually for each subject should be sufficient to discover subject-specific patterns that are customizsed according to the brain injury.

As for the limitations of the MSM, the computation of the posterior probability has a time complexity of *O*(*nm*^2^), where *m* is the number of latent variables, and *n* is the length of the sequence of the observed variable. This procedure has to be repeated for each training trial and each iteration during the optimization of the MSM's transition matrix. On the other hand, the logistic regression model is not time dependent, therefore the samples of log-transformed variance are only computed once. This results in a large difference of calibration time between the MSM and the sliding window logistic regression model: the MSM may take up to tens of seconds to converge, while training the logistic regression classifier is a matter of several hundredths of a second. However, such difference in calibration time may not be prohibitive in practice, since subjects are usually asked to rest for several minutes between runs.

The MSM and sliding window classifiers achieved classification performance levels (i.e., TPR, FPR) comparable to previous studies (Townsend et al., 2004; Muralidharan et al., 2011). However, a similar comparison with previous literature is more challenging with respect to the latency of detection. This is due to the fact that, in most asynchronous ERD-based BCI studies, latency or delay are regarded as the time required to achieve the peak classification performance between two classes (i.e left and right motor imagery) (Pfurtscheller et al., 2009).

The proposed model could be applied to phenomenons other than motor imagery, such as gait related ERD (Wagner et al., 2012; Lisi and Morimoto, 2015) or processing of sensory and cognitive information (Pfurtscheller and Lopes da Silva, 1999), as long as an abrupt change in power occurs in the signal of interest. The MSM could be extended to adaptively handle the non-stationarities of the EEG signal (Vidaurre et al., 2011), by means of Bayesian update techniques. It may also be possible to handle multi-class problems: for each additional class, a new hidden state should be included, and the Gaussian observation models should be parameterized with a covariance rather than a variance in order to handle multiple CSP projections. Given the Bayesian nature of the model, we could also include priors related to external environment, or to the current state of a neuroprosthesis in order to achieve context-dependent behavior.

## Author contributions

Conceived and designed the experiments: GL, AT, and JM. Performed the experiments: GL and AT. Analyzed the data: GL, DR, and AT. Contributed reagents, materials, analysis tools: GL, JM, and DR. Wrote the paper: GL. Drafted the article: GL. Revised the article: GL, DR, AT, and JM. Final approval: GL, DR, AT, and JM . Agreed to be accountable for all aspects of the work: GL, DR, AT, and JM.

### Conflict of interest statement

The authors declare that the research was conducted in the absence of any commercial or financial relationships that could be construed as a potential conflict of interest.
